# Ethrel‐Induced Enhancement of Sugar Accumulation and Postharvest Quality in Early‐Harvested Cantaloupe Melons

**DOI:** 10.1155/tswj/9929514

**Published:** 2026-01-29

**Authors:** Nidya Putri Zulia Kusuma Wardani, Ketty Suketi, Abdullah Bin Arif

**Affiliations:** ^1^ Agronomy and Horticulture Master′s Study Program, Postgraduate School, IPB University (Bogor Agricultural University), Bogor, Indonesia, ipb.ac.id; ^2^ Department of Agronomy and Horticulture, Faculty of Agriculture, IPB University (Bogor Agricultural University), Bogor, Indonesia, ipb.ac.id; ^3^ Research Center for Horticulture, National Research and Innovation Agency, Bogor, Indonesia, brin.go.id

**Keywords:** flesh color, postharvest, shelf life, storage, sugar content

## Abstract

Melon (*Cucumis melo L*. var. Cantaloupe) is classified as a climacteric fruit, which means its quality deteriorates quickly after harvest. Early‐harvested melons often exhibit inferior color quality and lower sugar content but have a longer shelf life than those harvested later. This study aims to improve the postharvest quality of early‐harvested cantaloupe melons by evaluating the effect of Ethrel treatment on their sweetness and overall quality throughout the storage period. The melons were immersed in an Ethrel solution at 0 (control), 25, 50, and 100 ppm concentrations. After treatment, the melons were air‐dried and stored in a controlled environment at 28^°^C ± 1^°^C with 80*%* ± 5*%* relative humidity for 21 days. The results indicated that treating the melons with Ethrel at a concentration of 25 ppm significantly enhanced their quality and shelf life. This concentration increased sweetness levels and sugar content (sucrose), which measured 2% higher than the other treatments after 7 days of storage. Additionally, the 25 ppm Ethrel treatment improved the melons′ color to a vibrant orange and helped retain their hardness, titratable acidity, and vitamin C content. Furthermore, this treatment resulted in minimal fruit damage and extended the melons′ shelf life for up to 21 days during storage. In conclusion, Ethrel at a concentration of 25 ppm is considered the optimal treatment for improving sugar content and maintaining the quality of early‐harvested cantaloupe melons.

## 1. Introduction

The melon, scientifically identified as *Cucumis melo* L., belongs to the Cucurbitaceae family and ranks the fifth most crucial fruit worldwide [1]. Melon displays genotypes and phenotypes grouped into intraspecific classifications [2]. Although melons come across multiple taste profiles [3, 4], many breeding programs focus on improving shelf life to increase market attractiveness, sometimes neglecting the importance of flavor quality [5–7]. One type of melon consumers favor is the cantaloupe melon (*Cucumis melo* L var. *Cantaloupe*). Consumers are inclined to choose cantaloupe melon for its pleasing and sweet taste. The pleasing sweetness of melon mostly comes from sucrose [8]. Moreover, melon is a climacteric fruit prone to postharvest damage [9, 10]. Melons spoil after about 10 days when kept in an ambient room [11].

Effective postharvest handling strategies for melon fruit are essential for superior quality and extended shelf life. Some tropical fruits, such as dragon fruit (*Hylocereus costaricensis*) and bananas (*Musa acuminata* L.) that are harvested early, have a longer shelf life and freshness compared to fruit harvested late because fruit harvested late quickly experiences a decline in quality [12–14]. Wardani et al. [15] reported that cantaloupe melons harvested early, specifically at 32 days after anthesis (DAA), 10 days earlier than the recommended harvest time, demonstrate a longer shelf life than fully ripe fruit. However, early‐harvested melons often have lower quality, particularly regarding sugar content, as their carbohydrates are primarily starch (polysaccharides) [15, 16]. Additionally, the flesh color of cantaloupe melons harvested early is paler than those harvested at full ripeness, making them less appealing to consumers [17]. Consumers prefer cantaloupe melons with attractive flesh color, appropriate moisture levels, sweetness, a crunchy texture, and a pleasing aroma [10]. Therefore, efforts are needed to improve the postharvest quality of melon fruit, especially sugar content and flesh color, and extend its shelf life.

An attempt to enhance the quality of melon fruit after harvesting involves ethylene. Melon ripening is characterized by intricate gene expression patterns and alterations in various physical and biochemical properties, including fruit firmness, color, soluble solids content, and ethylene production [11, 18]. Ethylene is crucial in the ripening process of climacteric fruits. Applying ethylene can improve the quality of climacteric fruits such as pears (*Pyrus communis*), melons, and kiwis (*Actinidia chinensis*) [19, 20]. Various postharvest treatments control the ripening of fruits by either enhancing or inhibiting ethylene production. Ethylene enhances the presence of carotenoids in melons, thereby developing appealing colors in the fruit flesh [21]. Gao et al. [22] reported the molecular mechanism underlying sucrose accumulation in melon fruit. Ethylene inhibits CmMYB44, a transcription factor that directly binds to the CmSPS1 and CmACO1 promoters to repress their expression. Meanwhile, ethylene induces CmERFI‐2, which in turn suppresses CmMYB44 transcription. This regulatory cascade ultimately increases melon fruit′s sucrose and ethylene levels. Further research is necessary to explore the potential of using ethylene to enhance the postharvest quality of early‐harvested melons. The right ethylene concentration is crucial for achieving the best possible fruit quality. Hence, the primary focus of the research was to enhance the postharvest quality of early‐harvested cantaloupe melons.

## 2. Materials and Methods

### 2.1. Plant Materials

Cantaloupe (*Cucumis melo* L. var. *Cantaloupe*) of the red aroma variety was cultivated in Jasinga, Bogor, Indonesia, under controlled conditions suitable for tropical climates, utilizing a fertigation system in a greenhouse setting. The greenhouse had a capacity for 400 cantaloupe plants. The melon fertilization procedure is presented in Table 1. Melon was harvested at 32 DAA, which is 10–12 days earlier than the harvesting time recommended by Agblor and Waterer [23] and Baraka et al. [24]. The melon fruits were transported 20 km to the laboratory and selected to obtain undamaged and uniform‐sized fruits. The fruits were rinsed with a sodium hypochlorite solution at 100 mL/L concentration.

**Table 1:** The melon fertilization procedure.

**Day after planting (DAP)**	**Fertilizer type**	**Composition/content**	**Concentration**	**Application method**	**Duration/notes**
0 (planting media preparation)	Base media	Soil, rice husks, and manure (2:1:1), CaCO_3_, SP36 phosphate, and trichoderma biofertilizer	‐ CaCO_3_: 150 g/m^2^ ‐SP36 phosphate: 30 g/m^2^ ‐Trichoderma biofertilizer: 10 g/m^2^	Mixed into planting media	Base medium preparation before planting
3 DAP	AB mix fertilizer	Fertilizer A: Calcium nitrate, Librel BMX; Fertilizer B: MgSO_4_, KNO_3_, KH_2_PO_4_, and Phytoplex	700 ppm	Fertigation	5 min
7 DAP	AB mix fertilizer	—	700 ppm	Fertigation	10 min
15, 27, 37, and 42 DAP	Supernanotech liquid fertilizer	—	1 mL/16 L water	Foliar spray	—
20 DAP	AB mix+NPK fertilizer	AB mix+NPK (16:16:16)	1200 ppm	Fertigation	10 min
35 and 40 DAP	AB mix fertilizer	—	1800 ppm	Fertigation	10 min
50, 52, 56, and 60 DAP	AB mix fertilizer	—	2000 ppm	Fertigation	10 min

### 2.2. Experimental Design, Ethrel, and Storage Treatments

The experiment was conducted using a completely randomized design (CRD). The treatment involved different concentrations of Ethrel 480 SL (C2H6ClO3P). The Ethrel concentrations tested were 0, 25, 50, and 100 parts per million (ppm). Ethrel was applied on the day of harvest by submerging cantaloupe melons in a solution at the designated concentrations for 1 min. One hundred twenty fruits were used, with approximately 30 fruits assigned to each Ethrel concentration, distributed across three replications (10 fruits per replication). Each experimental unit consisted of 10 fruits treated with a specific Ethrel concentration, which allowed for the assessment of both destructive and nondestructive parameters. Eight fruits were allocated per experimental unit for the destructive measurements to evaluate total soluble solids (TSS), sugar content, flesh color, softness, titratable acidity (TA), vitamin C content, and rind surface structure. Two fruits were reserved for nondestructive measurements, which included ethylene production, respiration rate, weight loss, and fruit damage. After treatment, all cantaloupe melons were stored at room temperature (25°C–28°C) with a relative humidity of 80*%* ± 5*%* for 21 days. Samples for destructive measurements were taken from two fruits in each experimental unit on Days 0, 7, 14, and 21; however, the assessment of rind surface structure was conducted only at the beginning and the end of the storage period. Nondestructive measurements were carried out on two sample fruits per experimental unit every 3 days throughout the storage duration.

### 2.3. Measurements of Ethylene Production and Respiration Rate

Measuring ethylene production and respiration rate refers to the research of Arif et al. [25], which was modified on abiu fruit. Measurement of ethylene production and respiration rate in cantaloupe melon fruit was done using a three‐gas analyzer (Merck Felix, type F‐950). Measurements were made by inserting two whole melons into a 6‐L closed jar for 30 min. The ethylene production was expressed as parts per million per kilogram per hour (ppm/kg h), and the respiration rate was expressed as milliliters per kilogram per hour (mL/kg h).

### 2.4. Measurements of TSS and Sugar Content

The TSS was measured from melon cantaloupe juice extracted in triplicate using the Atago DR‐A1 digital refractometer (Atago Co. Ltd., Tokyo, Japan) at 28^°^C ± 1^°^C according to the method suggested by Arif et al. [25]. The TSS values were reported in degrees Brix (°Brix).

Measurement of sugar contents (glucose, sucrose, and fructose) followed the method described by Susanto et al. [26] with slight modifications. Melon fruit samples (5 g) were homogenized with 20 mL of distilled water and sonicated for 30 min. The extract was then filtered through a microfilter and passed through a 0.45‐*μ*m syringe filter before analysis. Quantitative determination of glucose, fructose, and sucrose was performed using high‐performance liquid chromatography (HPLC) (Dionex Ultimate 3000 RS, Thermo Fisher Scientific, United States) equipped with a ZORBAX Carbohydrate Column (Agilent Technologies, California, United States) and a corresponding guard column. The mobile phase consisted of acetonitrile and water (75:25, *v*/*v*) under isocratic conditions at a 1.0 mL/min flow rate. The column temperature was maintained at 35°C, and the injection volume was 5 *μ*L. Detection and identification of individual sugars were based on retention times and calibration curves obtained from authentic standards, with determination coefficients (*R* ^2^) exceeding 0.999. The concentrations of glucose, fructose, and sucrose were expressed as percentages of fresh weight.

### 2.5. Measurements of Flesh Color

Melon flesh color measurement refers to research by R. Kasim and M. Kasim [27], which was measured using a chromameter (Model CR 400, Konica‐Minolta, Osaka, Japan). The chromameter was calibrated with a white field. Melon flesh is measured for *L* ^∗^ (brightness level), *a* ^∗^ (green [−] or [+]), and *b* ^∗^ (blue [−] to yellow [+]). The color of the melon rind and flesh is measured by attaching the lens of the chromameter to the melon rind. Color observations are measured from three sides of the melon (tip, middle, and base), and the average is determined.

### 2.6. Measurements of Softness and Weight Loss

The fruit softness was determined following the method developed by Sun et al. [28] with some modifications. The fruit softness was measured using a semiautomatic penetrometer. Measurements are made by inserting a penetrometer needle at four locations on the seam: The first and second were to close the flesh in the middle of the fruit. The third and fourth locations were on the melon tail, and the last four locations were on the fruit equator.

The weight loss was calculated using the method proposed by Arif et al. [25]. Fruit weight loss (%) was calculated according to the formula: weight loss (*%*) = ([weight before storage − weight after storage])/weight before storage) × 100*%*. The weight loss was expressed as a percentage (%).

### 2.7. Measurements of TA and Vitamin C

There are at least two organic acids that can be identified in cantaloupe, such as citric and malic acids [29]. The most dominant acid in most cantaloupes is malic acid [30]. The NaOH titration method described by Arif et al. [25] was used to determine the TA content. TA was determined by titrating the extract against 0.1 N NaOH and then expressed as percentage malic acid equivalents. The equivalent weight (EW) of acid included in the TA calculation formula of melon fruit is EW malic acid, with a value of 67. Based on Sadler and Murphy [31], the formula for calculating the TA content of cantaloupe is as follows:

TA %=volume NaOH mL×fp×0.1 N×EWsample weight g×100%.

The remarks are as follows:

fp = Dilution factor.

EW = equivalent weight (malic acid).

The vitamin C content was evaluated using the titration method suggested by Fitriana and Fitri [32]. To prepare the sample, the flesh of the cantaloupe melon was mashed until the juice was released, which was then filtered using a cloth filter. A total of 10 g of the extracted juice was placed into a 100‐mL measuring flask and diluted with distilled water to the mark. Then, 10 mL of this solution was transferred to an Erlenmeyer flask. Three drops of starch solution were added as an indicator, and the solution was titrated with iodine until the color changed to a stable dark blue. The iodine used as the titrant had a normality of 0.01 N. In calculating vitamin C content, the EW is based on the dominant acid present, ascorbic acid [33]. The EW of ascorbic acid is 0.88 g/eq. The vitamin C was expressed as milligrams per 100 g (mg/100 g).

### 2.8. Scanning Electron Microscopy Surface Structure of the Cantaloupe Rind

The surface structure of cantaloupe melon rind was observed, referring to the method carried out by Gautam et al. [34] on modified cantaloupe melon commodities. The fruit′s rind was cut into 0.5 × 1 × 2 cm^3^ sizes. The melon rind pieces were dried using a −55°C freeze dryer for 24 h. The cantaloupe rind was coated with Au using a sputter coater for 2 min. The Au‐coated samples were then observed at different magnifications using a scanning electron microscope (SEM) (Hitachi SU‐3500).

### 2.9. Fruit Damage

Cantaloupe fruit, during storage, is categorized as damaged if it is no longer suitable for marketing with symptoms such as bruising, base rot, or fungal growth. Fruit damage is calculated as a percentage by dividing the number of damaged fruits by the total number of fruits in the same treatment [35].

### 2.10. Data Analysis

Data were examined using a one‐way analysis of variance for each storage day. The significant differences among the treatment means were established by the Duncan multiple range test (DMRT) at a probability level of 5%. Statistical analyses of data were conducted using SAS Portable (v 9.1.3). The data were presented as the mean of three replications.

## 3. Result and Discussion

### 3.1. Ethylene Production and Respiration Rate

Ethylene is the dominant hormonal trigger for climacteric fruit ripening, and ethylene‐dependent and ethylene‐independent regulatory pathways coexist to coordinate the ripening process in melons. Thus, some physiological processes during ripening have been established to be ethylene‐dependent, while others are ethylene‐independent or very sensitive to low ethylene levels. In many fruits, such as melons, ripening is associated with a transient increase in respiration and autocatalytic ethylene production. Climacteric respiration, stalk abscission zone formation, rind yellowing, and rind carotenoid content in Charentais‐type cantaloupe melon (cv. Védrantais) are ethylene‐dependent processes [36, 37]. Membrane damage and volatile synthesis partially depend on ethylene [36, 37]. Ethrel treatment affected the respiration rate and ethylene production (Figure 1). The climacteric peak of cantaloupe melon treated with Ethrel at concentrations of 25 and 50 ppm occurred on the sixth day of storage, with ethylene production ranging from 2.40 to 4.43 ppm/kg h (Figure 1a). In contrast, fruit treated with 100 ppm Ethrel reached the climacteric peak on the 12th day of storage, producing 6.11 ppm/kg h of ethylene (Figure 1a). The control fruit exhibited its climacteric peak later, on the 15th day of storage, with ethylene production of 2.57 ppm/kg h (Figure 1a). In addition, in this study, applying Ethrel at 50 and 100 ppm on cantaloupe melon fruit showed higher respiration rates than the control and 25 ppm Ethrel treatments (Figure 1b). Ethrel treatment of 25 ppm increased ethylene production slightly, and the respiration rate was relatively low. Thus, a slight increase in ethylene can induce positive physiological changes to improve the quality of cantaloupe melon fruit without shortening the shelf life. Controlled ethylene levels can reduce respiration, delaying starch deterioration and resulting in more complex and less sweet fruits during storage, thus extending the shelf life [38, 39].

Figure 1Effect of Ethrel treatments on (a) ethylene production and (b) respiration rate in cantaloupe melons during storage. Data are means of three replicates. Different letters in the same day indicate a significant difference by the Duncan multiple range test (*p* < 0.05).

**Figure:**
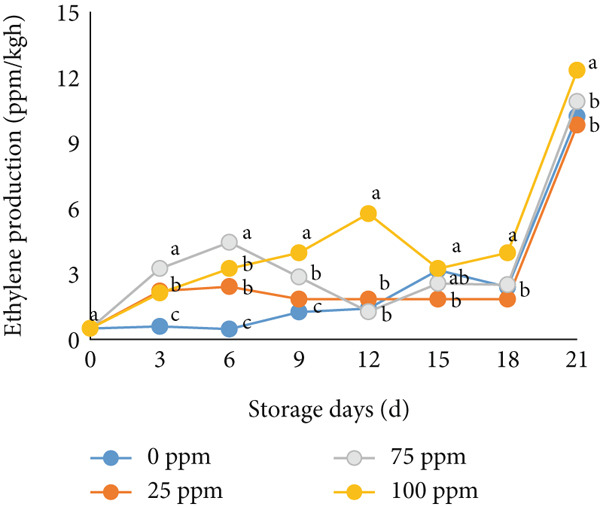
(a)

**Figure:**
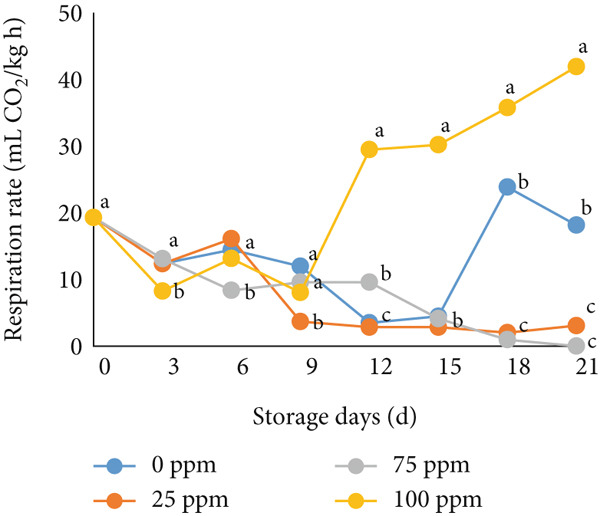
(b)

### 3.2. TSS and Sugar Content

TSS is a critical indicator of fruit quality and consumer acceptance [40]. In this study, both TSS and total sugar content in cantaloupe melon increased progressively during storage (Figure 2a,b). At the beginning of the 14‐day storage period, the melon fruits treated with Ethrel exhibited higher sugar content compared to the control, suggesting that Ethrel treatment accelerates sugar accumulation during ripening. The accumulation of sugars in Ethrel‐treated fruits occurred approximately 7–14 days earlier than in untreated fruits (Figure 2b). The total sugar content of the cantaloupe melon fruit in this study ranged between 2% and 5% (Figure 2b). For comparison, melon fruits at harvest have been reported to contain total sugar levels ranging from 23.23 to 61.40 mg/100 g [41]. Similar trends have been observed in other climacteric fruits; for instance, tomatoes show an increase of about 1.5% in total sugar content from the green to red ripening stages [42], while mangoes exhibit an increase of approximately 28.55% during storage [43].

Figure 2Effect of Ethrel treatments on (a) TSS, (b) total sugar, (c) sucrose, (d) fructose, and (e) glucose contents in cantaloupe melons during storage. Data are means of three replicates. Different letters in the same day indicate a significant difference by the Duncan multiple range test (*p* < 0.05).

**Figure:**
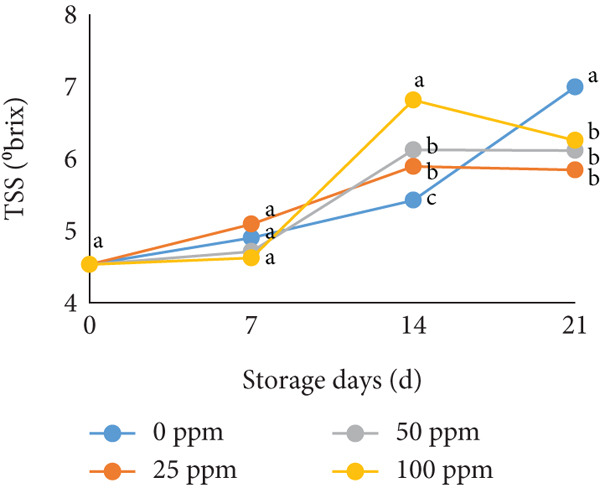
(a)

**Figure:**
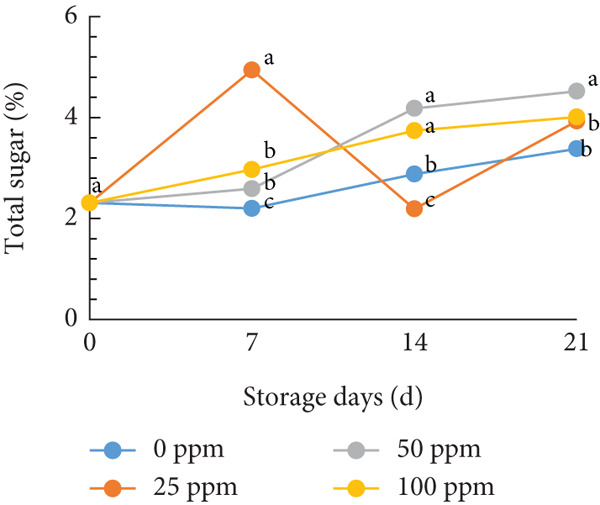
(b)

**Figure:**
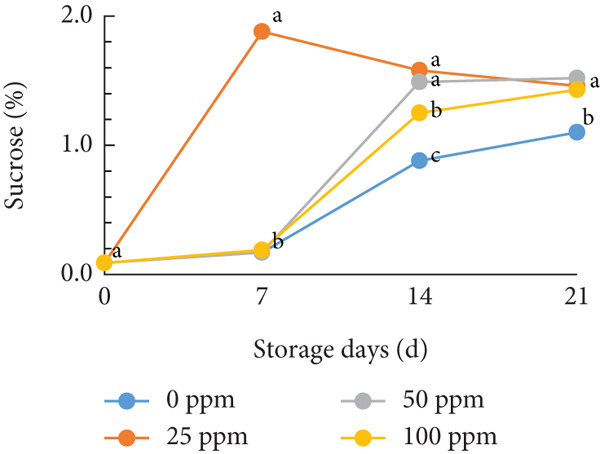
(c)

**Figure:**
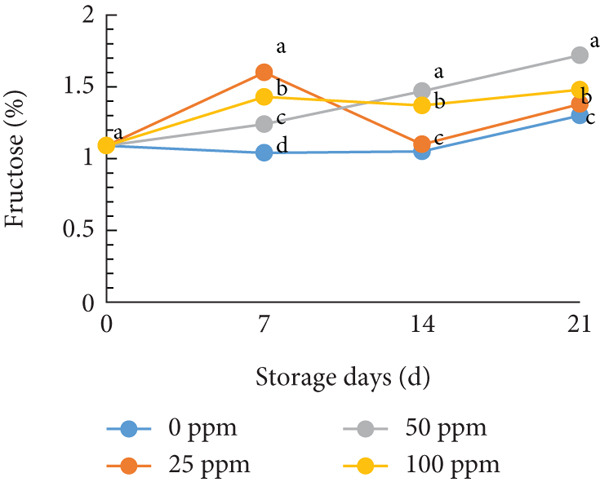
(d)

**Figure:**
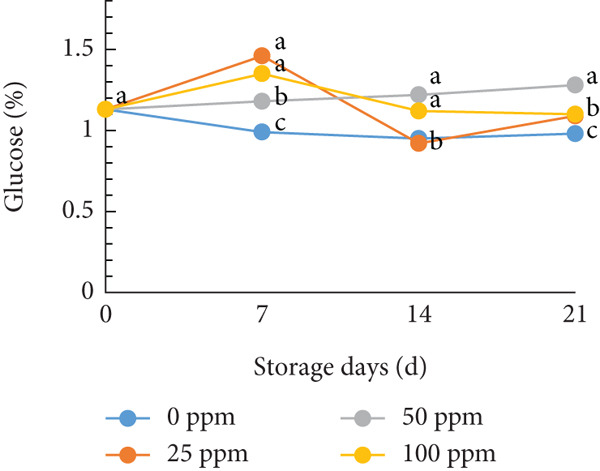
(e)

The main soluble sugars in ripe melon fruit are sucrose, glucose, and fructose [44]. Among these, sucrose is a crucial metabolic substrate, providing carbon skeletons and energy essential for plant growth and development [45]. Moreover, sucrose plays a significant role in determining the taste quality of melon fruit, as its accumulation directly contributes to the total sugar content and perceived sweetness [45, 46]. One of the key factors influencing sugar accumulation in fruit is ethylene, a plant hormone that regulates the expression of genes involved in sucrose and starch metabolism [47]. In the present study, Ethrel treatment at 25 ppm promoted a faster accumulation of sucrose in cantaloupe melon fruit compared to other treatments during storage (Figure 2c). This result aligns with the findings of Farcuh et al. [48], who reported that ethylene enhances sucrose accumulation by reducing sucrose catabolism and stimulating sucrose biosynthesis. Furthermore, exogenous ethylene treatment has been shown to increase both sucrose levels and ethylene production by improving the activities of key enzymes such as sucrose synthase (SS) and sucrose phosphate synthase (SPS) in climacteric melon fruit [49].

In cantaloupe melons, the accumulation of fructose and glucose during storage was lower than that of sucrose (Figure 2d,e). However, fruits treated with Ethrel exhibited higher levels of fructose and glucose compared to the control (Figure 2d,e). The application of 25 ppm Ethrel effectively accelerated the increase in fructose and glucose content, particularly evident 7 days after treatment. This finding suggests that Ethrel treatment enhances sugar metabolism, contributing to improved sweetness and overall sugar content of the fruit during storage. Ethylene released from Ethrel functions as a signaling molecule that regulates carbohydrate metabolism during fruit ripening. According to Lao et al. [50], ethylene promotes the accumulation of glucose and fructose in melon fruit. In the mesocarp tissue of melon, sucrose represents approximately 58.1% of total soluble sugars, while glucose and fructose contribute 17.5% and 25.6%, respectively [51]. The relatively lower proportion of glucose and fructose compared to sucrose reflects the typical sugar composition of ripe melon fruit, as fructose predominantly accumulates during early fruit development and is later converted into sucrose during ripening [52, 53]. During storage, the increase in glucose concentration is generally less pronounced than that of sucrose. Lao et al. [50] reported that sucrose hydrolysis into glucose and fructose in melon is catalyzed by the enzyme invertase. This enzymatic process occurs during ripening, where both acid and neutral invertases cleave sucrose—a disaccharide—into its constituent monosaccharides, glucose and fructose, which are subsequently utilized for energy metabolism and storage. The ethylene‐mediated activation of sugar‐metabolizing enzymes, therefore, explains the elevated levels of glucose and fructose observed in Ethrel‐treated cantaloupe melons.

### 3.3. Flesh Color

Typically, fruit color is a key attribute influencing consumer preference, as it serves as a visual indicator of ripeness and quality. During the ripening process, pigmentation changes in both the skin and flesh determine the final color of the fruit [54, 55]. In cantaloupe melon, the brightness value (*L* ^∗^) of the flesh gradually decreased during storage (Figure 3a). This reduction in the *L* ^∗^ value indicates the progression of ripening, where the flesh color becomes deeper as the fruit matures. Among the treatments, the melon fruit treated with 100 ppm Ethrel exhibited the lowest *L* ^∗^ value after 7 days of storage, signifying more advanced ripening compared to other treatments. Conversely, the redness (*a* ^∗^), yellowness (*b* ^∗^), and chroma values showed a significant increase, a trend that was also observed in fruits treated with 25 ppm Ethrel (Figure 3). The *L* ^∗^ parameter represents lightness, where a value of 0 denotes black and 100 denotes white [56, 57]. As shown in Figure 3a, fruits treated with 0 and 50 ppm Ethrel maintained relatively high *L* ^∗^ values for up to 7 days of storage, followed by a gradual decline, indicating increasing fruit maturity [58]. In the study of R. Kasim and M. Kasim [27], the reduction in the *L* ^∗^ value after several days of storage is attributed to pigment transformation in the melon flesh, resulting in a shift from bright green‐yellow‐orange hues to darker shades characteristic of ripe fruit.

Figure 3Effect of Ethrel treatments on (a) lightness (*L* ^∗^), (b) redness (*a* ^∗^), (c) yellowness (*b* ^∗^), and (d) chroma in cantaloupe melons during storage. Data are means of three replicates. Different letters in the same day indicate a significant difference by the Duncan multiple range test (*p* < 0.05).

**Figure:**
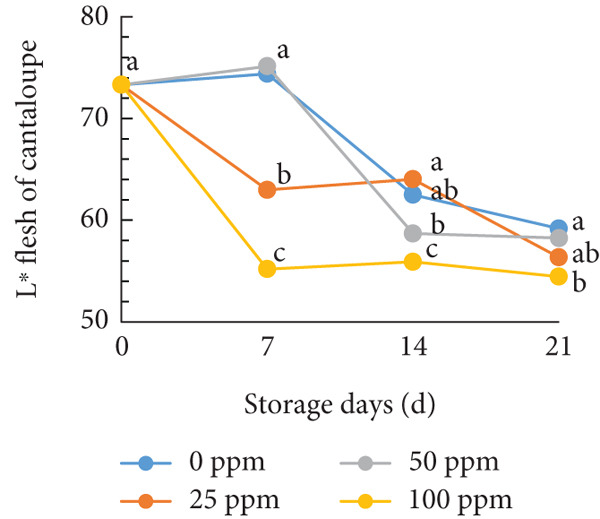
(a)

**Figure:**
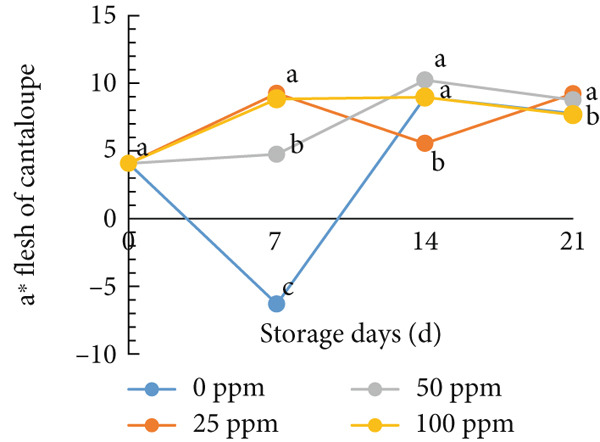
(b)

**Figure:**
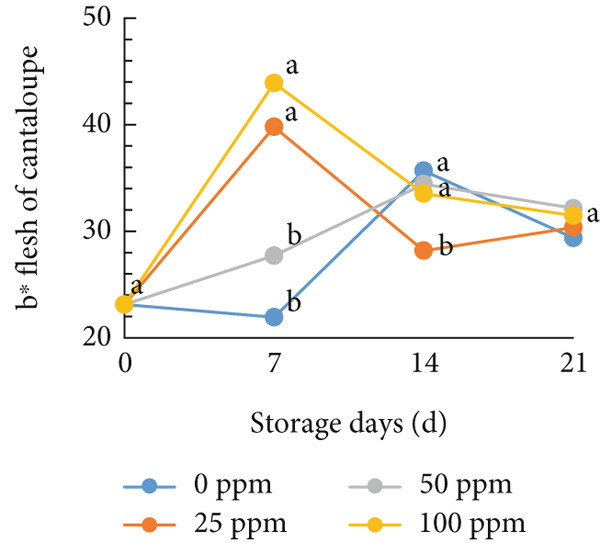
(c)

**Figure:**
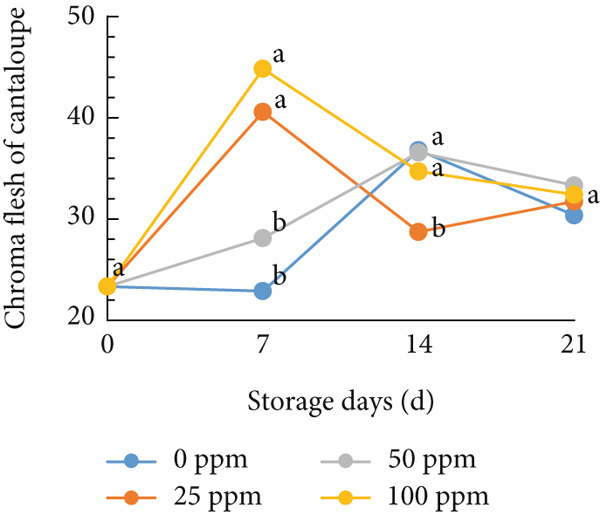
(d)

Furthermore, the redness (*a* ^∗^) value of cantaloupe melon treated with Ethrel at concentrations of 25 and 100 ppm showed a significant increase on the seventh day of storage (Figure 3b). This indicates that Ethrel application at these concentrations can effectively enhance the development of red or orange pigmentation in the fruit flesh. Higher ethylene concentrations accelerate color changes in fruit flesh toward an orange hue [59]. This study′s application of Ethrel at 25 and 100 ppm also resulted in the highest yellowness (*b* ^∗^) values after 7 days of storage (Figure 3c). Endogenous ethylene produced by the fruit is crucial in the color transition of climacteric fruit flesh during storage [60, 61]. Adding exogenous ethylene may stimulate endogenous ethylene biosynthesis, amplifying its physiological effects. Introducing exogenous ethylene to melon fruit can accelerate tissue softening, enhance endogenous ethylene production, and hasten ripening [60]. Moreover, exogenous ethylene application in climacteric fruits is reported to reduce the green pigmentation of both the flesh and skin [61]. In this study, the Ethrel treatments at 25 and 100 ppm also exhibited the highest chroma values after 7 days of storage (Figure 3d). Chroma represents the intensity or purity of a color hue [62]. Conversely, in the control treatment, the chroma value of cantaloupe melon flesh increased only after 14 days of storage. In the study of Munira et al. [63], the chroma value of cantaloupe melon flesh diminished after 2 weeks of storage.

### 3.4. Softness and Weight Loss

As one of the fundamental parameters determining postharvest quality, fruit tenderness significantly influences both shelf life and consumer acceptance. Ethylene plays a crucial role in fruit softening and loss of firmness by activating cell wall–degrading enzymes such as polygalacturonase, which hydrolyzes pectin within the cell wall, leading to tissue softening. Excess ethylene exposure negatively affects fruit firmness and shortens shelf life, making fruits softer and more perishable [64]. The decrease in melon fruit firmness is typically associated with ripening and a substantial rise in endogenous ethylene production [65]. The observed increase in fruit softness during storage is consistent with the stimulation of ethylene biosynthesis, which enhances softening as ethylene concentration increases [66, 67]. In this study, the tenderness of cantaloupe melon flesh did not differ significantly among treatments after 7 days of storage. However, a gradual increase in tenderness was observed toward the end of the storage period (Figure 4a). Interestingly, melons treated with 25 ppm Ethrel exhibited slightly higher firmness than other treatments (Figure 4a).

Figure 4Effect of Ethrel treatments on (a) softness and (b) weight loss in cantaloupe melons during storage. Data are means of three replicates. Different letters in the same day indicate a significant difference by the Duncan multiple range test (*p* < 0.05).

**Figure:**
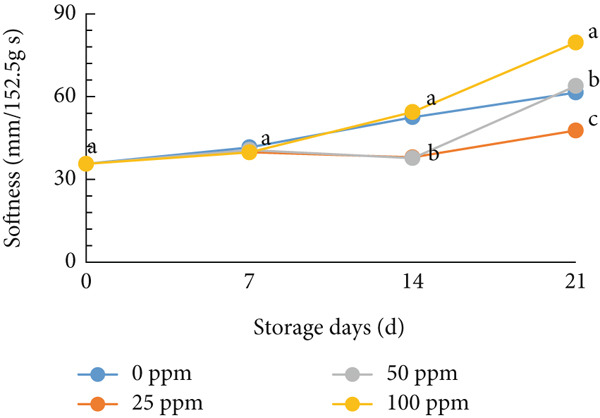
(a)

**Figure:**
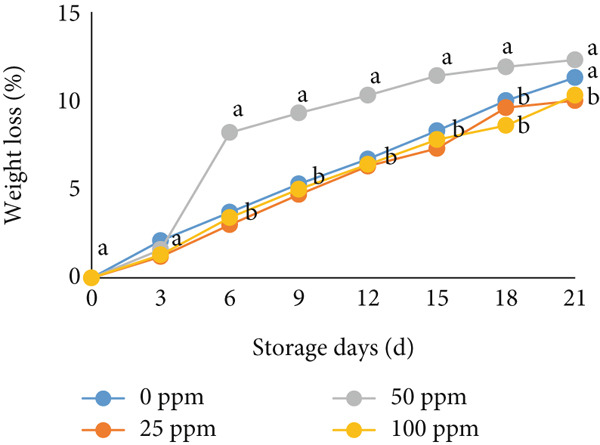
(b)

Furthermore, weight loss is another critical parameter related to fruit shelf life, affecting visual quality and marketability [68]. In this study, weight loss in cantaloupe melons increased progressively during storage (Figure 4b), although it remained below 20%, which is considered relatively low. The application of exogenous ethylene can accelerate the breakdown of cell wall membranes and covalently bound pectins, promote the conversion of pectin into water‐soluble forms, and hasten the degradation of hemicellulose and cellulose, thereby contributing to fruit softening and weight loss [69–72].

### 3.5. TA and Vitamin C

The TA and vitamin C are also important markers of fruit quality. As shown in Figure 5, TA in melon fruit tended to decrease during storage for all treatments. However, the 25 ppm Ethrel and control substantially inhibited the loss of TA up to 14 days of storage. Melon fruit′s TA primarily reflects its organic acid content. These acids are gradually degraded through respiration or converted into sugars and volatile compounds during ripening. Thus, the decline in TA indicates the catabolism of organic acids that occurs concurrently with sugar accumulation. Ethylene acts as the key hormone regulating fruit ripening by activating enzymes in organic acid metabolism and converting these acids into intermediates for sugar synthesis and respiration. However, the effect of ethylene is highly concentration‐dependent. A moderate ethylene level, such as that induced by Ethrel at 25 ppm, can stimulate ripening without excessively accelerating respiration, leading to a slower decline in TA. The 25 ppm Ethrel treatment likely generates sufficient ethylene to enhance sugar accumulation—through increased activities of enzymes such as invertase and SS—while not being high enough to hasten acid degradation. Consequently, this produces a balanced ripening process, characterized by elevated sugar levels and a relatively slow decrease in acidity, maintaining a higher TA value during storage. During respiration, organic acids in tomato are converted into sugars, and their derivatives or use during storage can be factors causing TA decline [25]. TA increased when entering the early ripening phase and decreased at the end of ripening [73]. Furthermore, Cocco et al. [67] reported that adding exogenous ethylene can reduce TA in climacteric fruit; the higher the ethylene concentration, the faster the fruit ripening process, thus accelerating the accumulation of water and sugar in the fruit. In this study, low‐concentration Ethrel treatment effectively suppressed the reduction of TA in early‐harvested cantaloupe melons. The increase in ethylene production, which tends to be small in the 25 ppm Ethrel in cantaloupe melons, is expected to increase sugar content and slightly decrease TA during ripening.

Figure 5Effect of Ethrel treatments on (a) TA and (b) vitamin C in cantaloupe melons during storage. Data are means of three replicates. Different letters in the same day indicate a significant difference by the Duncan multiple range test (*p* < 0.05).

**Figure:**
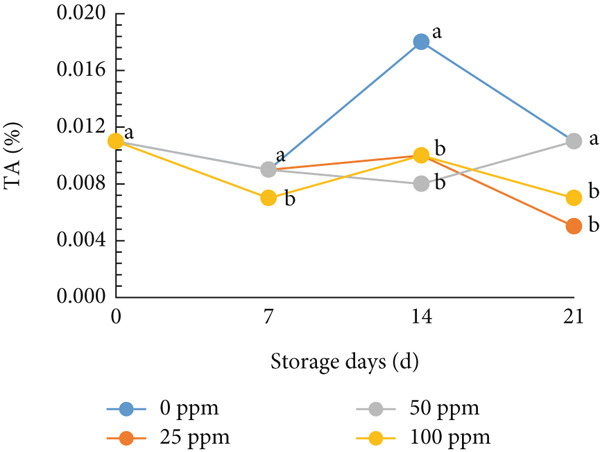
(a)

**Figure:**
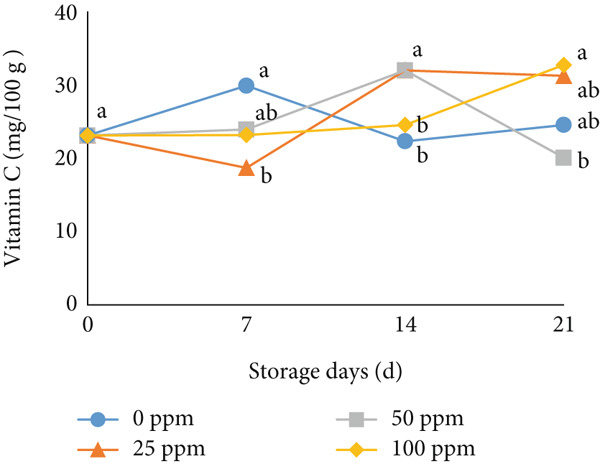
(b)

In addition to TA, vitamin C is an essential nutrient susceptible to oxidation during storage [74]. Vitamin C is an important antioxidant that can delay senescence and reduce reactive oxygen species [75]. Vitamin C levels in early‐harvested cantaloupe melons decreased during the storage period for all treatments (Figure 5b). However, applying 25 ppm on postharvest of cantaloupe melons, which were harvested early, showed a higher vitamin C content and could be maintained during storage. Therefore, applying 25 ppm Ethrel on the postharvest of cantaloupe melons can maintain TA and vitamin C content during storage.

### 3.6. Structure of Cantaloupe Rind Intercellular Space Number and Size

The amount of intercellular space increased after 14 days of storage (Figure 6a). Although the increase was not statistically significant, the trend indicated that fruits treated with 50 and 100 ppm Ethrel exhibited a pronounced increase in intercellular spaces toward the end of the storage period. Similarly, the size of the intercellular spaces began to increase after 7 days of storage (Figure 6b). A significant enlargement was observed on Day 14 in fruits treated with 50 ppm Ethrel, followed by a reduction in intercellular space size at 21 days of storage.

Figure 6(a) Structure of cantaloupe rind intercellular space number and (b) intercellular space size. Data are means of three replicates. Different letters in the same day indicate a significant difference by the Duncan multiple range test (*p* < 0.05).

**Figure:**
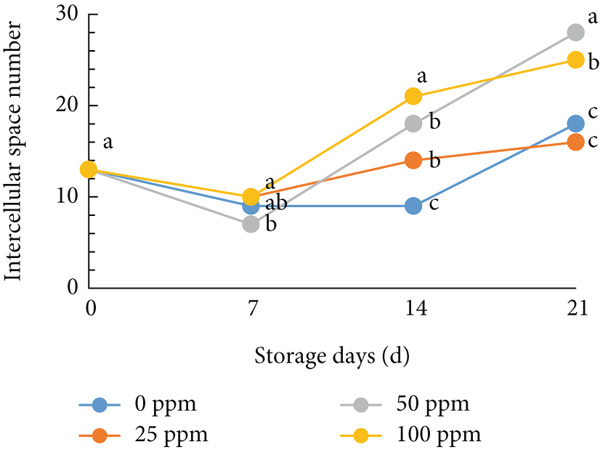
(a)

**Figure:**
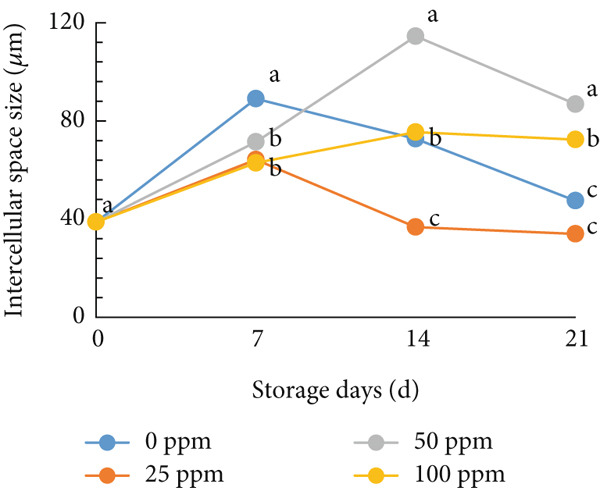
(b)

Fallik et al. [76] observed that many intercellular spaces were formed after 2 weeks of storage, which caused the epidermis of the fruit rind to open so that transpiration took place quickly and resulted in decay. Transpiration occurs in fruits and vegetables through the epidermis wax layer and the rind′s pores (stomata and lenticels). Transpiration mainly occurs through the intercellular space in the tissue [77]. The intercellular space is interconnected with narrow capillary tubes, providing an efficient means of “ventilation” in the fruit tissue [78].

The extent of tissue degradation increases significantly with the level of ripeness, as fruit softening leads to the formation of intercellular spaces. As ripening progresses, cell wall degradation becomes more pronounced. Figure 7 illustrates the changes in intercellular spaces observed during 21 days of storage under different Ethrel concentration treatments. The increasingly wide intercellular spaces are due to the accumulation of pectin substances, particularly in the middle lamella and intercellular regions, which facilitates the separation of cell walls and expansion of cell‐to‐cell connections. Pereira et al. [78] reported that postharvest changes in rind structure are closely associated with reduced intercellular bonds in papaya fruit (*Carica papaya* L.) during storage. Similar patterns of cell wall degradation and increased intercellular spacing have also been documented during the ripening of apple (*Malus domestica*) [79], persimmon (*Diospyros kaki* Thunb. cv. Rojo Brillante) [80], and papaya (*Carica papaya* L.) [78].

Figure 7SEM images of cantaloupe melon rind treated with Ethrel at (a) 0 ppm, (b) 25 ppm, (c) 50 ppm, and (d) 100 ppm after 21 days of storage.

**Figure:**
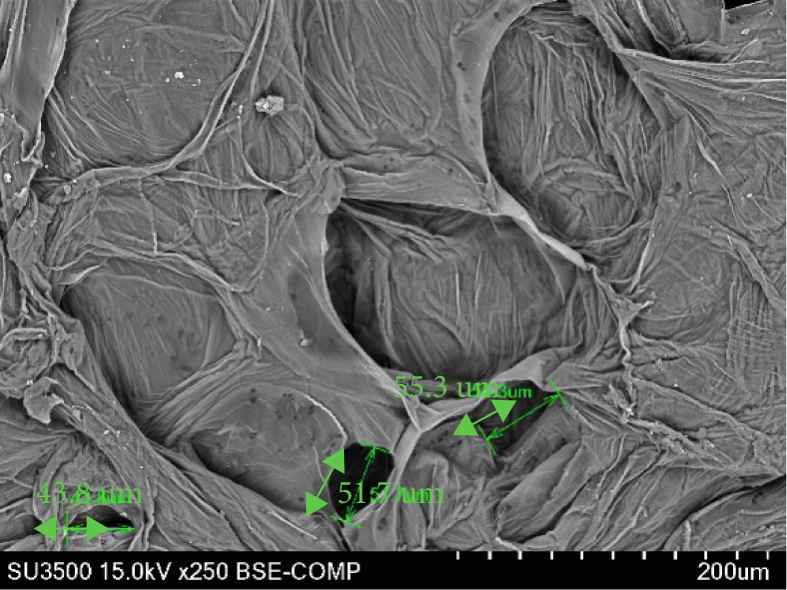
(a)

**Figure:**
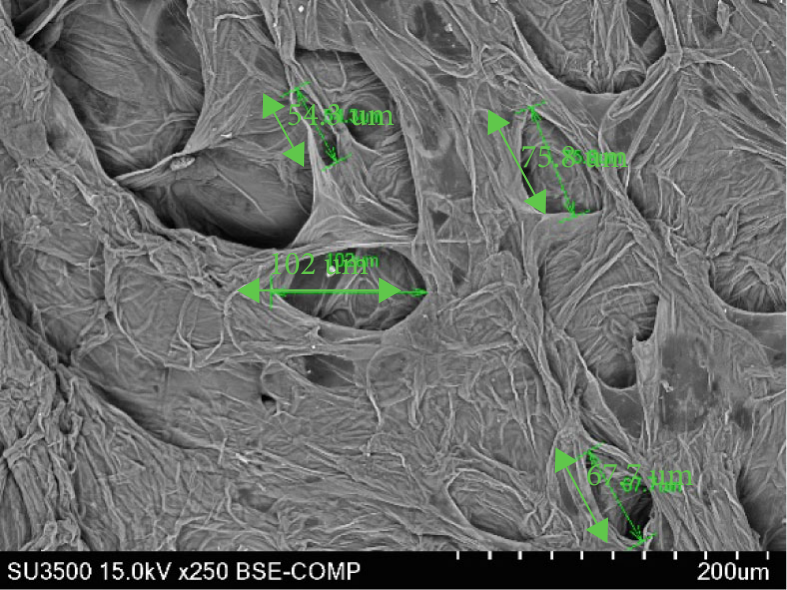
(b)

**Figure:**
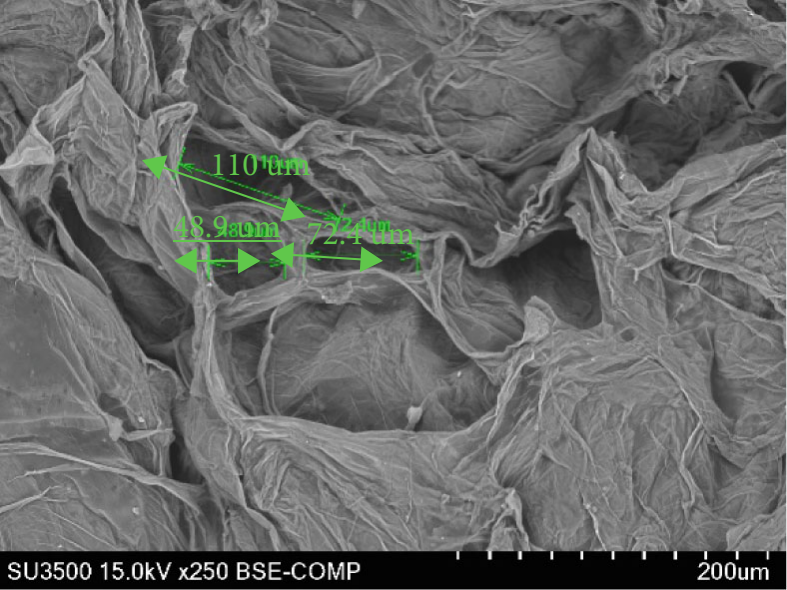
(c)

**Figure:**
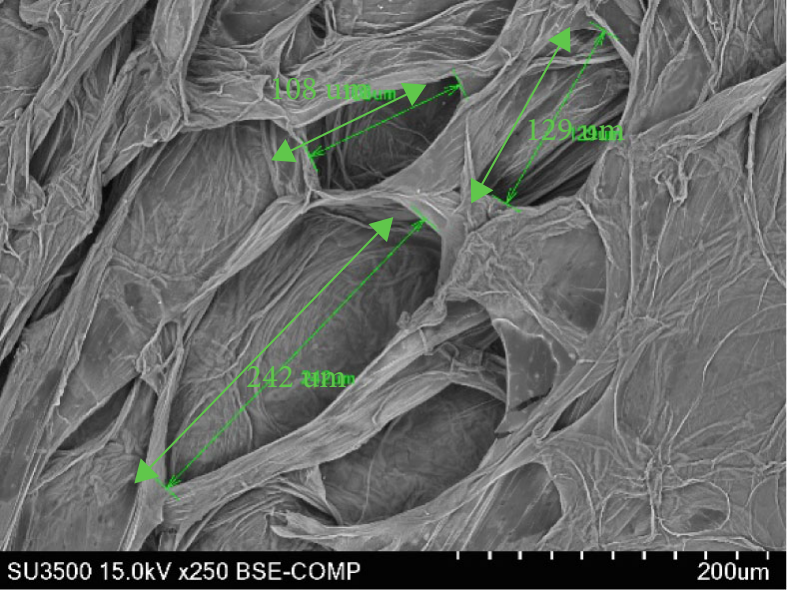
(d)

Intercellular spaces are formed during fruit storage due to increased fruit cell volume and decreased cell adhesion [81]. The size of the intercellular space increases during ripening due to the degradation of the middle lamella and cell membranes with starch hydrolysis [82–84]. The increase in the size of the intercellular space does not induce an increase in diffusivity because it can be filled with fluid due to cell leakage. The intercellular space as a means of tissue aeration depends on the total volume and its continuity filled with gas or liquid [85]. During storage, the size of the cell space decreases, presumably because the fruit loses moisture through evaporation. This loss of moisture causes the cells to become drier, and the intercellular space shrinks. Cells in the cantaloupe rind can also shrink or wrinkle over time due to water loss, making the intercellular space smaller.

### 3.7. Fruit Damage

**Figure 8:**
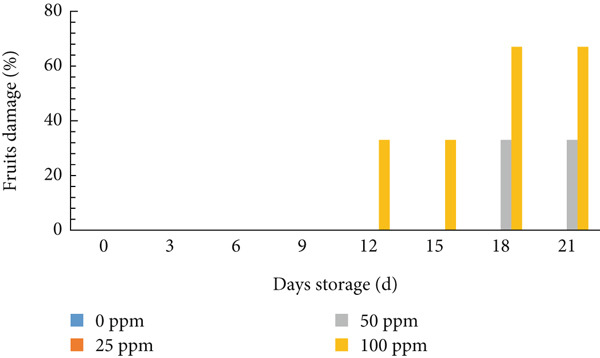
Percentage of cantaloupe melon fruit damage during storage.

Melon fruit damage occurred faster in the 100 ppm Ethrel treatment, with damage symptoms starting at 12 days of storage and 50 ppm at 18 days of storage (Figure 8). The results of fruit damage observations align with ethylene production; the higher the ethylene concentration, the faster the fruit damage occurs. Melon fruit damage during storage is influenced by ethylene production during storage. Storing climacteric fruit without storage at low temperatures can increase ethylene production so that the fruit ages quickly [61, 86].

## 4. Conclusion

Ethrel treatment at 25 ppm improves early‐harvested cantaloupe melons′ quality and shelf life. The treatment of 25 ppm Ethrel increased the sweetness level and sugar content (sucrose), which was 2% higher than other treatments at 7 days of storage. In addition, applying 25 ppm Ethrel also increased the color to orange and maintained hardness, TA, and vitamin C. Furthermore, applying 25 ppm Ethrel showed low fruit damage and extended the shelf life of melons up to 21 days of storage. Therefore, the 25 ppm Ethrel is considered the appropriate treatment to increase sugar content and maintain the quality of early‐harvested cantaloupe melons.

## Conflicts of Interest

The authors declare no conflicts of interest.

## Funding

This study was funded by the Kementerian Pendidikan, Kebudayaan, Riset, dan Teknologi, 10.13039/501100023174, 22262/IT3.D10/PT.01.03/P/B/2024.

## Data Availability

The data that support the findings of this study are available from the corresponding author upon reasonable request.
